# Risk-Stratified Cardiovascular Screening Including Angiographic and Procedural Outcomes of Percutaneous Coronary Interventions in Renal Transplant Candidates

**DOI:** 10.1155/2014/854397

**Published:** 2014-06-19

**Authors:** Julian König, Martin Möckel, Eda Mueller, Wolfgang Bocksch, Seema Baid-Agrawal, Nina Babel, Ralf Schindler, Petra Reinke, Peter Nickel

**Affiliations:** ^1^Department of Cardiology, Charité Campus Virchow-Klinikum, Augustenburger Platz 1, 13353 Berlin, Germany; ^2^Department of Nephrology and Intensive Care, Charité Campus Virchow-Klinikum, Augustenburger Platz 1, 13353 Berlin, Germany; ^3^Division of Emergency Medicine, Charité Campus Virchow-Klinikum and Mitte, Augustenburger Platz 1, 13353 Berlin, Germany; ^4^Department of Cardiology and Angiology, Charité Campus Mitte, Charitéplatz 1, 10117 Berlin, Germany; ^5^Berlin-Brandenburg Center for Regenerative Therapies (BCRT), Charité Campus Virchow-Klinikum, Charite-Universitätsmedizin Berlin, Augustenburger Platz 1, 13353 Berlin, Germany

## Abstract

*Background*. Benefits of cardiac screening in kidney transplant candidates (KTC) will be dependent on the availability of effective interventions. We retrospectively evaluated characteristics and outcome of percutaneous coronary interventions (PCI) in KTC selected for revascularization by a cardiac screening approach. *Methods*. In 267 patients evaluated 2003 to 2006, screening tests performed were reviewed and PCI characteristics correlated with major adverse cardiovascular events (MACE) during a follow-up of 55 months. *Results*. Stress tests in 154 patients showed ischemia in 28 patients (89% high risk). Of 58 patients with coronary angiography, 38 had significant stenoses and 18 cardiac interventions (6.7% of all). 29 coronary lesions in 17/18 patients were treated by PCI. Angiographic success rate was 93.1%, but procedural success rate was only 86.2%. Long lesions (*P* = 0.029) and diffuse disease (*P* = 0.043) were associated with MACE. In high risk patients, cardiac screening did not improve outcome as 21.7% of patients with versus 15.5% of patients without properly performed cardiac screening had MACE (*P* = 0.319). *Conclusion*. The moderate procedural success of PCI and poor outcome in long and diffuse coronary lesions underscore the need to define appropriate revascularization strategies in KTC, which will be a prerequisite for cardiac screening to improve outcome in these high-risk patients.

## 1. Introduction

In recent years, many end stage renal disease (ESRD) patients with advanced age or significant cardiovascular disease are accepted on the growing waiting lists because of the survival benefit kidney transplantation may confer even to high risk patients [1–6].

As kidney transplant candidates frequently have severe coronary artery disease (CAD) and a high cardiovascular mortality, invasive or noninvasive screening for CAD and revascularization in case of significant myocardial ischemia have long been recommended [7–10]. However, since randomized controlled studies in nonrenal populations showed no benefit of preoperative revascularization [11, 12], nowadays revascularization is recommended only in patients with high risk coronary lesions and significant symptoms and/or ischemia [13].

Furthermore, current guidelines in the general population recommend basing revascularization strategies in complex CAD on coronary lesion characteristics, since the SYNTAX trial demonstrated that complex coronary lesions were associated with worse outcome of PCI compared to coronary artery bypass grafting (CABG) [13, 14]. In ESRD patients, however, little is known about the optimal strategy in treating complex coronary lesions. On the one hand, PCI in ESRD patients is technically challenging due to the frequently complex and severely calcified coronary lesions [15–18]. On the other hand, CABG has been associated with increased mortality compared to nonrenal patients [18].

To our knowledge no single study has reported till date lesion and procedural characteristics of PCI performed during cardiac evaluation of kidney transplant candidates. In addition, it is noteworthy that waiting times, risk factors such as ethnicity [5, 6, 19], and practice patterns of cardiac screening [20] in kidney transplant candidates show large international variations but have been reported mostly from Northern and South America [21–29], and there is paucity of comparable European data [4, 30–32].

Therefore, we describe the characteristics and outcome of PCI in patients selected for revascularization by a cardiovascular screening approach from a cohort of 267 renal transplant candidates evaluated at our center between 2003 and 2006. Our data underscore the need to address complexity of coronary lesions in future studies that evaluate cardiac screening approaches to define appropriate interventions in kidney transplant candidates.

## 2. Subjects and Methods

### 2.1. Patients and Study Groups

All patients who were referred for renal transplant wait listing to our center between January 2003 and December 2006 were screened for inclusion (*n* = 574). Patients who were evaluated externally or with incomplete data were excluded. Cardiovascular screening procedures performed until wait listing were reviewed in detail by one investigator (JK). In patients with PCI, coronary angiograms were reviewed by an experienced cardiologist (MM) to specify lesion characteristics and types according to the American Heart Association/American College of Cardiology (AHA/ACC) classification as well as angiographic success [33]. Both investigators were blinded to patients' outcome. MACE and death from all causes were assessed by review of medical records and data bases. In addition, between December 2009 and June 2010 all patients or, in case of a fatal event, relatives and dialysis centers were interviewed by telephone for MACE occurrence. Mean follow-up time was 55.3 ± 19.3 months after wait listing.

Our prespecified protocol of basic cardiac investigation was comprised of a 12-lead resting electrocardiogram (ECG) and a transthoracic echocardiography. Based on estimated clinical risk and functional status patients were referred for ergometry and/or stress echocardiography. In addition, patients with poor functional status or inconclusive ergometry result were referred for dobutamine stress echocardiography and/or coronary angiography. The final decision which patient was referred for a stress test and/or a coronary angiography was at the discretion of the attending cardiologist or nephrologist. For the study, every patient was retrospectively classified as high or low risk based on the American Society of Transplantation guidelines [8] and the work of Kasiske et al. [23]: high risk was defined by diabetes, history of ischemic heart disease, and/or 2 of the following risk factors: age over 50, current smoker, hypertension, peripheral vascular disease, or history of a cerebrovascular disease. A stress test was considered conclusive when target frequency was reached and/or ischemia was found. Cardiac screening was defined as properly performed if high risk patients had a conclusive stress test and/or a coronary angiography before wait listing. CAD was defined by history of myocardial infarction or cardiac intervention, for example, CABG or PCI. Peripheral vascular disease was defined by history of limb amputation or revascularization. Significant coronary artery disease was defined as coronary artery stenosis of ≥50%. In the analysis, only the first baseline cardiac screening tests performed until wait listing were included.

### 2.2. End Points of the Study

Primary endpoint was the composite incidence of fatal or nonfatal MACE defined by myocardial infarction, revascularization procedures (CABG/PCI), sudden death, and ischemic stroke occurring after wait listing. Secondary endpoint was death from all causes after wait listing.

In patients with PCI, angiographic success was defined as achievement of a TIMI flow grade 3 and final residual stenosis <25% per lesion, using any percutaneous method. Procedural success was defined as angiographic success without the occurrence of MACE during 30 days after intervention.

### 2.3. Statistical Analyses

Statistical analysis was performed with PASW statistics 18.0. Differences between groups were assessed using Mann Whitney *U* test for continuous variables and Chi-square, Fisher's exact, or Kruskal Wallis tests, as indicated for categorical variables. Patient survival after wait listing was estimated using the Kaplan-Meier product limit method, and curves were compared using the log-rank test. Univariate and multivariate stepwise backward Cox regression analyses were performed to identify predictors of cardiovascular events after wait listing. As 96.6% of all patients were hypertensive, hypertension was not included into this analysis. A multivariate stepwise backward logistic regression model was used to identify predictors of death from all causes.

## 3. Results

### 3.1. Patient Characteristics and Screening Tests before Wait Listing

Of 574 patients originally referred for transplant evaluation, 267 patients were included who received cardiac evaluation directly at our center. Baseline clinical parameters are shown in Table 1(a), stratified according to the outcome. Mean age was 49 years, 26% were diabetics, and 18% had a history of CAD.

Cardiovascular screening procedures are shown in Table 1(b) according to the outcome (MACE) and in Table 2 according to the cardiovascular risk status at the evaluation time. A conclusive stress test was performed in 60% of high-risk and 52% of low-risk patients, showing significant ischemia in 25 high-risk and 3 low-risk patients (*P* = 0.033), which was followed by coronary angiography in 27/28 cases. Patients who underwent only treadmill ergometry compared to patients who underwent stress echocardiography had comparable age, gender, and smoking status, but less often a history of CAD (7% versus 19.8%; *P* = 0.053) and a significantly lower time on dialysis (12.4 versus 23.4 months; *P* = 0.035). One low-risk patient with positive ergometry testing was not referred to coronary angiography as he had negative stress echocardiography.

Only 24/41 patients with MACE and 130/226 patients without MACE had a conclusive stress tests at baseline (Table 1(b)). The sensitivity of noninvasive stress testing for predicting future MACE was 33.3%, the specificity was 84.6%, the positive predictive value was 28.6%, and the negative predictive value was 87.3%. Only 2/14 patients with ergometry and MACE had a conclusive stress test. Taken together, the predictive value of noninvasive screening was poor, as stress testing failed to identify 2/3 of the patients with future MACE due to the low sensitivity of the noninvasive testing. However, in the unadjusted Cox regression analysis (Table 4) it was shown that patients with positive stress testing still had a 2.79-fold increased risk for MACE.

**Figure 1:**
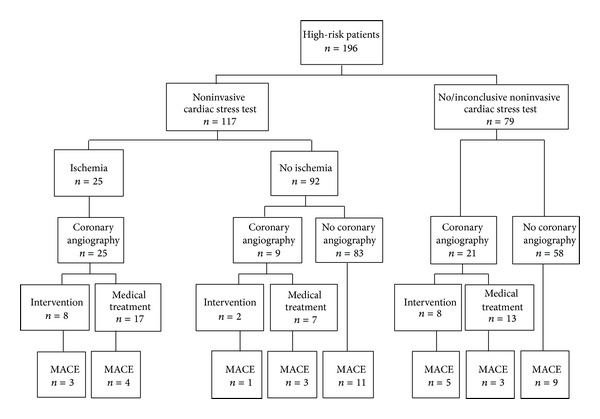
Cardiac evaluation procedures and cardiac events during followup in high-risk patients.

Altogether, coronary angiography was performed in 58 patients (94.8% high-risk patients), revealing significant coronary artery stenoses in 38 of 58 (65.5%) patients.  Figure 1 shows cardiac screening procedures along with MACE during followup in high-risk patients. In the 9 patients without ischemia in noninvasive stress testing referral for coronary angiography might have been due to abnormal ECG in 1 patient, resting wall motion abnormalities in 4 patients, and stable angina in 2 patients as additional risk factors. In 2 patients, the reason for coronary angiography remained unknown. Of 21 patients with no or inconclusive noninvasive stress test, 11 had angina, 3 showed resting wall motion abnormalities, 3 had known CAD and/or PVD, 1 had poorly controlled diabetes, and 1 patient was excessive smoker. The exact reason for coronary angiography in 2 other patients remained unknown.

In 21 patients with positive stress test and/or angina no coronary intervention was performed. In 11 of these patients coronary stenoses without significant ischemic or perfusion area were found. In 8 of these patients no significant coronary stenoses were found at all, and in 2 patients intervention was not performed for high risk coronary lesions and recurrent gastrointestinal bleeding, respectively.

Notably, 58 of the 196 high-risk patients were left without properly performed cardiac screening, that is, without a conclusive stress test or a coronary angiography. However, during follow up, in this group only 9/58 (15.5%) patients experienced MACE which was comparable to a total of 30 MACE (21.7%) in the 138 patients who underwent a properly performed cardiac screening prior to active wait listing (*P* = 0.319, Figure 1).

### 3.2. Cardiac Interventions before Wait-Listing

18 patients (6.7% of all included patients, all high risk) were offered coronary revascularization according to ACC/AHA guidelines [33]. No patient refused. All patients who underwent cardiac intervention had evidence of cardiac ischemia in prior stress testing and/or angina. One patient with severe 3-vessel disease and proximal left major coronary artery stenosis was referred to CABG without prior PCI. Two other patients were ultimately referred to CABG after PCI. Altogether, 29 coronary lesions in 17 patients were treated by PCI including 29 stents. Complete revascularization, defined as successful treatment of all lesions in major epicardial coronary vessels by PCI, was achieved in 8 of the 17 patients (47.1%) who underwent PCI. 3 patients were ultimately completely revascularized by CABG.

### 3.3. Baseline Angiographic and Procedural Characteristics

Table 3(a) shows baseline angiographic characteristics of the 29 PCI in 17 patients, comparing those with MACE versus those without MACE after wait listing. Of note, long lesions (*P* = 0.029) and diffuse disease (*P* = 0.043) were significantly more common in patients with MACE than in those without. Furthermore, stent length was significantly higher in long lesions >20 mm compared to shorter lesions (mean length 22.6 versus 15 mm; *P* = 0.005) but was not significantly different in diffuse versus nondiffuse disease lesions (mean length 21.6 versus 18.9 mm; *P* = 0.419, not shown). No difference was found in the number of stents per lesion between lesions >20 mm and shorter lesions (mean stents per lesion 1.06 versus 0.96; *P* = 0.614) as well as between diffuse and nondiffuse disease lesions (both groups on average 1 stent per lesion; *P* = 0.519). Calcification grades were not different between patients with or without MACE (*P* = 0.988).

Table 3(b) shows procedural characteristics of the 29 PCI in 17 patients, comparing those with MACE versus those without MACE during followup. Neither the number of stents implanted nor stent length was different between these two groups. Angiographic success rate in all patients was 93.1%. During 30 days after first PCI, 2 MACE were observed, which were a re-PCI for in-stent restenosis at day 25 in one patient and acute stent thrombosis at day 5 after the first PCI in another patient, lowering the procedural success rate to 86.2% in all patients.

### 3.4. MACE and Deaths after Wait Listing

41 patients (15.4%) had at least one MACE after wait listing (39 high-risk patients; *P* = 0.001; Table 2). First MACE were coronary (re)interventions in 18 (43.9%), myocardial infarction in 13 (31.7%), ischemic stroke in 6 (14.6%), and sudden death in 4 (9.8%) cases. 11 (26.8%) events were fatal.

133 of 267 patients ultimately received a kidney transplant from deceased (*n* = 91) or living donors (*n* = 42) during followup. Only 6 MACE were observed after transplantation (4.5%), which were myocardial infarctions at 48, 416, and 772 days after transplant, respectively, 2 cardiac reinterventions at 973 and 1418 days, and 1 sudden death at 1190 days after transplant.

Causes of the 51 deaths observed after wait listing were sepsis in 19 (37.3%), cancer in 9 (17.6%), myocardial infarction in 6 (11.8%), sudden death and stroke each in 4 (7.8%), cerebral haemorrhage in 2 (3.9%), heart failure and pulmonary embolism each in 1 (2%), and other in 5 (9.8%) patients.

### 3.5. Predictors of MACE after Wait-Listing

Table 1 shows bivariate comparison of multiple parameters in patients with MACE versus without MACE. Age, diabetes, history of coronary, cerebrovascular and peripheral vascular disease (and as a consequence also high-risk status), and the duration of dialysis before wait listing were all significantly higher in patients with MACE. Patients who experienced MACE had significantly more ischemia in noninvasive stress testing (*P* = 0.034), had significantly more often been referred for coronary angiography (*P* < 0.001), and had more significant coronary artery stenoses (*P* = 0.005) compared to those without MACE.

Table 4 shows unadjusted HR for predictors of MACE in Cox regression analyses. When the 6 baseline parameters of the 267 patients with *P* < 0.01 in the univariate analysis of Table 4 were included in a multivariate model, only age, history of coronary artery disease, history of cerebrovascular disease, and time on dialysis before wait listing were predictors of MACE.

## 4. Discussion

This comprehensive analysis evaluated PCI characteristics and outcome in kidney transplant candidates selected for revascularization by a risk-stratified screening approach as performed in everyday practice before wait listing. While cardiac screening resulted in a low coronary intervention rate of 6.7%, we found that PCI in these selected high risk patients was only moderately effective and treatment of longer and diffuse disease coronary lesions was associated with increased risk of MACE. In line with current recommendations in nonrenal populations, our data underscore the need to address complexity of coronary lesions when revascularization strategies are investigated in kidney transplant candidates [13, 14].

Interestingly, we found a significantly higher stent length used in the treatment of longer lesions as a possible contributing cause for lower PCI effectivity in our study, as higher stent length has previously been associated with increased risk of restenosis both in BMS and drug eluting stents (DES) [34, 35].

In the few patients treated with DES, we found a trend for lower rate of MACE compared to patients treated with BMS. However, while DES have become the treatment of choice in the majority of PCI procedures with superior results even in ESRD patients due to lower rates of target lesion revascularization compared to BMS [36, 37], current European Guidelines for Myocardial revascularization recommend no universal use of DES in ESRD patients, as DES have not been shown to be of general advantage compared to BMS in these patients, and end stage renal disease is a risk factor for potentially fatal late stent thrombosis [38]. In addition, DES placement may delay transplantation due to the need of prolonged dual antiplatelet medication.

ESRD patients have been previously reported to have a higher risk of incomplete revascularization after PCI and higher procedural failure rates [39–43]. On the other hand, CABG in ESRD patients was associated in prior studies with a 3-fold greater perioperative mortality compared to nonrenal patients [18]. Nevertheless, a large recent USRDS analysis of almost 22,000 dialysis patients who underwent multivessel coronary revascularization reported a significantly lower risk for death and myocardial infarction with CABG compared to PCI [44]. However, diffuse disease is a well-known therapeutic challenge for both PCI and coronary artery bypass grafting, and a recent study showed acceptable results also with the use of multiple overlapping DES in the treatment of diffusely diseased LAD vessels [45], which might open up new treatment options also for ESRD patients. We agree with the recommendations of ESC guidelines on myocardial revascularization, which recommend PCI in patients with poor general condition for lower in-hospital mortality and complication rates of cardiac intervention, while CABG would preferentially be recommended in younger patients with good clinical condition for better long-term event-free survival [38].

Further risk factors for MACE were identified in our study. In line with previous observational studies, the clinical risk stratification was closely associated with MACE, as more than 95% of MACE occurred in high-risk patients [4, 19, 22, 23, 28–32]. Moreover, the performances of a positive stress test and a coronary angiography were each associated with MACE. Different screening approaches have been used in renal transplant candidates: Based on concerns regarding low sensitivity of stress tests in ESRD, some groups perform a coronary angiography in every patient prior to wait listing [26, 27, 46]. However, most of these studies did not indicate the details of the PCI. For instance, only the study by Kumar et al. indicated whether the PCI performed in 117 of 657 screened patients were done with or without stenting [46].

While current guidelines in nonrenal patients recommend screening only in patients with new or worsening cardiac symptoms or with a poor functional capacity [45], a recent AHA/ACCF scientific statement recommended cardiac screening in patients with no active cardiac conditions based on the presence of multiple CAD risk factors regardless of functional status [10]. In our study, the everyday-practice cardiac screening approach allowed us to apply retrospectively a risk stratification for comparing outcome in patients who underwent properly performed cardiac screening versus patients without. We found that outcome was not improved with properly performed screening based on the risk stratification used. While other risk stratifications remain be investigated and a confounding bias cannot be excluded due to the retrospective study design, the lack of correlation with outcome might also be related to ineffective revascularization by PCI in complex coronary lesions performed in our study.

However, our data do not support the view that cardiac screening is not warranted in kidney transplant candidates. Most importantly, ESRD patients without active cardiac conditions have a high incidence of CAD with prognostic relevance which should be diagnosed to know the extent of CAD and start medical treatment early. In line with previous studies, we found simple risk stratification based on comorbidities to be useful in the prediction for CAD and cardiovascular events, which should be used to guide stress testing for ischemia and consecutive referral for coronary angiography. Stress testing may identify areas of ischemia to guide interventions in coronary stenoses.

In prior studies, risk-stratified screening resulted in variable coronary angiography rates of 5–51% [4, 22, 23, 27, 29, 30, 32], and the rate of 20.2% reported in our study lies approximately in the middle. PCI rates in these studies were generally low between 1 and 5.7%. The PCI rate reported in our study (6.7%) is slightly higher, which might be related to the high proportion (73%) of high-risk patients and a long average time on dialysis of 26.7 months before wait listing.

With follow-up times between 2 and 7.4 years, the overall incidence of MACE has been reported in previous studies between 2.9 and 13% [22, 23, 29, 30, 32]. Corresponding to the risk profile and high rate of coronary angiographies in our patients, we found a higher incidence (15.4%) of MACE during a mean follow-up period of 55 months. Another reason for this may have been the accuracy of our data collection and great efforts spent on identification of each MACE including telephone interviews.

Strengths of our study include thorough collection of comorbid conditions and outcome analysis including telephone interviews. However, several limitations have to be acknowledged. The observational character of the study may be associated with selection bias and confounding. For instance, the exact criteria used for referring individual patients for stress testing and coronary angiography could not be identified retrospectively in each patient. The screening approach may be significantly improved when dobutamine stress echocardiography would be used in every patient. Furthermore, the number of patients undergoing PCI was small. Clearly, our results need to be confirmed in larger prospective studies. While a strategy of systematically screening and treating significant CAD as demonstrated by Kumar et al. resulted in high patient survival rates, our data emphasize the potential risks associated with the performance of PCI in complex CAD [46]. Therefore, we suggest that randomized clinical trials assessing effectivity of preoperative cardiac evaluation of renal transplant candidates that have already been proposed [47] should base revascularization strategies on assessments of coronary lesion characteristics.

In conclusion, the moderate procedural success rate of PCI and poor outcome in long and diffuse stenoses underscore the need to address coronary lesion characteristics to define appropriate revascularization strategies in kidney transplant candidates. Effective coronary interventions should be the basis for future randomized trials that investigate whether cardiac screening improves outcome.

## Figures and Tables

**Table:** (a) Baseline characteristics

Variable	Total (*n* = 267)	Patients without MACE (*n* = 226)	Patients with MACE (*n* = 41)	*P* value∗
**High risk (%)**	196 (73.4)	157 (69.5)	39 (95.1)	**0.001**
**Age (year)**	49.3 ± 13.4	48 ± 13.7	56.4 ± 8.9	**<0.001**
Previous renal transplant (%)	54 (20.2)	45 (19.9)	9 (22.0)	0.765
**Smoking (%)**	117 (43.8)	92 (40.7)	25 (61)	**0.016**
Male (%)	177 (66.3)	147 (65.0)	30 (73.2)	0.311
**Diabetes (%)**	68 (25.5)	50 (22.1)	18 (43.9)	**0.003**
Hypertension (%)	258 (96.6)	219 (96.9)	39 (95.1)	0.561
**History of CAD (%)**	47 (17.6)	30 (13.3)	17 (41.5)	**<0.001**
**History of CVD (%)**	11 (4.1)	5 (2.2)	6 (14.6)	**<0.001**
**History of PVD (%) **	31 (11.6)	19 (8.4)	12 (29.3)	**<0.001**
Statin (%)	108 (40.4)	91 (40.3)	17 (41.5)	0.876
Renal replacement therapy				
HD (%)	225 (84.3)	187 (82.7)	38 (92.7)	0.120
PD (%)	21 (7.9)	18 (8.0)	3 (7.3)	
Preemptive (%)	21 (7.9)	21 (9.3)	0	
BMI (kg/m^2^)	24.9 ± 4.9	24.9 ± 4.9	24 ± 7.4	0.743
**Mean time on dialysis before WL (mo)**	26.7 ± 41.8	23.7 ± 36.2	43 ± 62.6	**0.002**
**Mean followup after waitlisting **	55.3 ± 19.3	57.0 ± 17.7	45.8 ± 24.3	**0.004**
Original renal disease				
Glomerulonephritis	89 (33.3)	—	—	
Polycystic	39 (14.6)	—	—	
Diabetic nephropathy	32 (12.0)	—	—	
Vascular/hypertension	27 (10.1)	—	—	
Unknown	27 (10.1)	—	—	
Other	25 (9.4)	—	—	
Reflux/pyelonephritis	17 (6.4)	—	—	
Interstitial nephritis	8 (3.0)	—	—	
Cancer	3 (1.1)	—	—	
**Deaths**	51 (19.1)	31 (13.7)	20 (48.8)	**<0.001**

**Table:** (b) Baseline cardiac screening/intervention

Variable	Total (*n* = 267)	Patients without MACE (*n* = 226)	Patients with MACE (*n* = 41)	*P* value∗
Echocardiography (%)				
LV-Hypertrophy (%)	181 (67.8)	155 (68.6)	26 (63.4)	0.615
Septum diameter (mm)	13.7 ± 2.1	13.7 ± 2.1	13.8 ± 2.3	0.954
LV ejection fraction (%)	59.2 ± 5.7	59.6 ± 5.1	58.0 ± 1.1	0.133
Noninvasive stress test (%)	204 (76.4)	173 (76.5)	31 (75.6)	0.896
Conclusive test (%)	154 (57.7)	130 (57.5)	24 (58.5)	0.786
Stress echocardiography (%)	122 (45.7)	98 (43.4)	24 (58.5)	0.073
Conclusive test (%)	111 (41.6)	89 (39.4)	22 (53.7)	0.896
Treadmill ergometry (%)	115 (43.1)	101 (44.7)	14 (34.1)	0.210
**Conclusive test (%)**	51 (19.1)	49 (21.7)	2 (4.9)	**0.016**
**Positive stress test**	28	20	8	**0.034**
**(% of all/of conclusive tests)**	(13.7, 18.2)	(11.6, 15.4)	(25.8, 33.3)	
**Coronary angiography (%)**	58 (21.7)	38 (16.8)	20 (48.8)	**<0.001**
** Significant coronary**				
**Artery stenosis (%)**	38 (66.7)	22 (57.9)	16 (80)	**0.005**
1-V-disease	15 (26.3)	12 (31.6)	3 (15)	
2-V-disease	11 (19.3)	3 (7.9)	8 (40)	
3-V-disease	12 (21.1)	7 (18.4)	5 (25)	
Revascularization (%)	18 (31.6)	9 (23.7)	9 (47.4)	0.070
**Stress test and/or CA (%)**	220 (82.4)	181 (80.1)	39 (95.1)	**0.020**
Conclusive stress test and/or CA (%)	176 (65.9)	144 (63.7)	32 (78)	0.075

*P value for comparison between patients without and with MACE.

CAD: coronary artery disease; CVD: cerebrovascular disease; HD: hemodialysis; PD: peritoneal dialysis; PVD: peripheral vascular disease; CA: coronary angiography; noninvasive stress test: stress echocardiography and/or treadmill ergometry.

**Table 2:** Baseline cardiac screening procedures and subsequent MACE in renal transplant candidates stratified according to their risk status.

Variable	Total (*n* = 267)	Low risk (*n* = 71)	High risk (*n* = 196)	*P* value∗
Echocardiography (%)				
LV-hypertrophy	181 (81.9)	49 (77.8)	132 (83.5)	0.315
LV ejection fraction (%)	59.2 ± 5.7	60.2 ± 4	58.8 ± 6.3	0.171
Septum diameter (mm)	13.72 ± 2.1	13.5 ± 2.2	13.8 ± 2	0.205
Noninvasive stress test (%)	204 (76.4)	56 (78.8)	148 (75.5)	0.567
Conclusive Test (%)	154 (57.7)	37 (52.1)	117 (59.7)	0.054
Stress Echocardiography (%)	122 (45.7)	27 (38)	95 (48.5)	0.130
Conclusive Test (%)	111 (41.6)	24 (33.8)	87 (44.4)	0.667
Treadmill ergometry (%)	115 (43.1)	37 (52.1)	78 (39,8)	0.073
Conclusive Test (%)	51 (19.1)	14 (19.7)	37 (18.9)	0.333
**Stress test positive (%)**	28 (13.7)	3 (5.4)	25 (16.9)	**0.033**
**Coronary angiography (%)**	58 (21.7)	3 (4.2)	55 (28.1)	**<0.001**
**Sign. coronary stenosis (%)**	38 (65.5)	0 (0)	38 (69.1)	**0.014**
Revascularization (%)	18 (31)	0 (0)	18 (32.7)	0.233
Stress test and/or coronary angiography (%)	220 (82.4)	57 (80.3)	163 (83.2)	0.585
**Conclusive stress test and/or coronary angiography (%)**	176 (65.9)	38 (53.5)	138 (70.4)	**0.010**
**Cardiovascular Event (%)**	41 (15.5)	2 (2.8)	39 (19.9)	**0.001**

*P values for comparison between low-risk and high-risk patients.

CAD: coronary artery disease; CVD: cerebrovascular disease; HD: hemodialysis; PD: peritoneal dialysis; PVD: peripheral vascular disease; CA: coronary angiography; noninvasive stress test: stress echocardiography and/or treadmill ergometry.

**(a):** 

Variable	Patients without MACE (*n* = 8)	Patients with MACE (*n* = 9)	*P* value
Total lesions	12	17	
Number of diseased vessels			0.664
1-Vessel disease	1	0	
2-Vessel disease	2	5	
3-Vessel disease	5	4	
Lesion vessel (>20 mm)			0.741
LAD	6	7	
LCX	3	6	
RCA	3	3	
Venous graft	0	1	
AHA/ACC lesion type			0.255
A	1	2	
B1	4	1	
B2	4	6	
C	3	8	
TIMI flow grade pre-PCI			0.678
Grade 0	1	1	
Grade 2	0	1	
Grade 3	11	15	
**Long lesion (>20 mm)**	4	13	**0.029**
Ostial lesion	2	7	0.234
Calcification			0.988
No	4	6	
Mild	6	8	
Severe	2	3	
**Diffuse disease**	1	8	**0.043**

LAD: left anterior descending coronary artery; LCX: left circumflex coronary artery; RCA: right coronary artery; TIMI: thrombolysis In myocardial infarction.

**(b):** 

Variable	Patients without MACE (*n* = 8)	Patients with MACE (*n* = 9)	*P* value
Total lesions	12	17	
Number of lesions treated per patient			0.590
1	5	4	
2	2	2	
3	1	3	
Number of stents per patient	1.5 (0–3)	1.89 (1–5)	0.618
(Mean, range)			
Stent length (mm)	16 (8–32)	23 (13–32)	0.263
(Median, range)			
Postdilatation	3	8	0.273
TIMI flow grade post PCI			0.414
Grade 0	1	0	
Grade 3	11	17	
Rotablator	0	3	0.246
CTO	1	1	1.000
Contrast volume per patient	243 (150–370)	300 (220–490)	0.200
(Mean, range)			
DES	3	1	0.279
BMS	9	16	0.365
Direct stenting	5	5	0.694
Angiographic success	11/12 (91.7%)	16/17 (94.1%)	
Procedural success	11/12 (91.7%)	14/17 (82.4 %)	

CTO: chronic total obstruction; TIMI: thrombolysis in myocardial infarction; DES: drug eluting stent; BMS: bare metal stent.

**Table 4:** Unadjusted and adjusted HR for predictors of MACE after wait listing.

Variable	Unadjusted hazard ratio		Adjusted hazard ratio^a^	
	*P* value	HR (95% CI)	*P* value	HR (95% CI)
*Patient characteristics *				
** Highrisk **	**0.004**	8.16 (1.97–33.79)		
** Age **	**<0.001**	1.05 (1.03–1.08)	**0.003**	1.05 (1.02–1.08)
Previous transplantation	0.694	1.16 (0.55–2.43)		
** Smoking**	**0.021**	2.10 (1.12–3.93)		
Gender	0.335	1.41 (0.70–2.80)		
** Diabetes **	**0.002**	2.61 (1.41–4.85)		
** History of CAD**	**<0.001**	4.32 (2.30–8.08)	**0.042**	2.09 (1.03–4.24)
** History of CVD**	**<0.001**	4.94 (2.07–11.77)	**0.018**	2.96 (1.20–7.31)
** History of PVD **	**<0.001**	4.20 (2.13–8.28)		
BMI (kg/m^2^)	0.153	0.95 (0.88–1.02)		
** Time on dialysis before wait listing**	**0.001**	1.01 (1.00-1.01)	**0.004**	1.01 (1.00-1.01)

** Screening/intervention **				
LV hypertrophy	0.735	0.87 (0.38–2.00)		
**Positive stress test **(28 of 154 conclusive stress tests)	**0.018**	2.79 (1.19–6.53)		
**Significant coronary artery stenosis** (38 of 58 coronary angiographies)	**0.039**	1.49 (1.02–2.17)		
Coronary intervention (PCI or CABG; 18 of 58 coronary angiographies)	0.202	1.78 (0.74–4.29)		

^a^Final model determined by Cox regression with stepwise selection.

CAD: coronary artery disease; CVD: cerebrovascular disease; PVD: peripheral vascular disease; LV hypertrophy: left ventricular hypertrophy; BMI: body mass index.
